# HKF-SVR Optimized by Krill Herd Algorithm for Coaxial Bearings Performance Degradation Prediction

**DOI:** 10.3390/s20030660

**Published:** 2020-01-24

**Authors:** Fang Liu, Liubin Li, Yongbin Liu, Zheng Cao, Hui Yang, Siliang Lu

**Affiliations:** 1 College of Electrical Engineering and Automation, Anhui University, Hefei 230601, China; ufun@ahu.edu.cn (F.L.); 15056086408@139.com (L.L.); caozheng@ahu.edu.cn (Z.C.); 15086@ahu.edu.cn (H.Y.); lusliang@mail.ustc.edu.cn (S.L.); 2 National Engineering Laboratory of Energy-Saving Motor and Control Technology, Anhui University, Hefei 230601, China

**Keywords:** rolling bearing, performance degradation, hybrid kernel function, krill herd algorithm, SVR

## Abstract

In real industrial applications, bearings in pairs or even more are often mounted on the same shaft. So the collected vibration signal is actually a mixed signal from multiple bearings. In this study, a method based on Hybrid Kernel Function-Support Vector Regression (HKF–SVR) whose parameters are optimized by Krill Herd (KH) algorithm was introduced for bearing performance degradation prediction in this situation. First, multi-domain statistical features are extracted from the bearing vibration signals and then fused into sensitive features using Kernel Joint Approximate Diagonalization of Eigen-matrices (KJADE) algorithm which is developed recently by our group. Due to the nonlinear mapping capability of the kernel method and the blind source separation ability of the JADE algorithm, the KJADE could extract latent source features that accurately reflecting the performance degradation from the mixed vibration signal. Then, the between-class and within-class scatters (SS) of the health-stage data sample and the current monitored data sample is calculated as the performance degradation index. Second, the parameters of the HKF–SVR are optimized by the KH (Krill Herd) algorithm to obtain the optimal performance degradation prediction model. Finally, the performance degradation trend of the bearing is predicted using the optimized HKF–SVR. Compared with the traditional methods of Back Propagation Neural Network (BPNN), Extreme Learning Machine (ELM) and traditional SVR, the results show that the proposed method has a better performance. The proposed method has a good application prospect in life prediction of coaxial bearings.

## 1. Introduction

Roller bearings are key components of rotating machinery and they are widely used in aerospace, railway and other industries [1]. Economic losses and major safety accidents can be avoided in industry through an accurate evaluation of the bearing degradation status of the equipment and a timely detection of bearing failures [2]. Two issues are key in the performance degradation evaluation of rolling bearings. One is to extract the performance degradation indicators [3] and the other is to establish effective prediction models [4]. Performance degradation index extraction is essential for bearing performance degradation assessment. In current studies, kurtosis, root mean square and peak indicators are used as indicators of bearing performance degradation [5]. However, completely reflecting the entire degradation process of the bearing using a single indicator parameter is difficult. Therefore, multi-domain features are extracted from the time domain and frequency domain. Then, these features are fused to remove the redundant features and used to characterize the bearing degradation process [6]. This process facilitates bearing degradation evaluation. Feature fusion techniques are generally divided into linear and nonlinear feature fusion methods [7]. Given that the vibration signal of the bearing is usually nonlinear, the nonlinear method has unique advantages in the fusion of bearing features [8]. For example, Zhang et al. used the Kernel Principal Component Analysis (KPCA) algorithm to fuse features [9].

Recently, a new algorithm named Kernel Joint Approximate Diagonalization of Eigen-matrices (KJADE) is invented by our group for feature fusion. This method is a combination of the kernel method and the traditional JADE algorithm [10]. Due to the nonlinear mapping capability of the kernel method and the blind source separation ability of the JADE algorithm. KJADE could extract latent source features that accurately reflecting the performance degradation from the mixed vibration signal.

On the other hand, an effective prediction model is critical to accurate performance evaluation [11]. In recent years, data-driven prediction models have been widely applied to performance degradation assessment [12]. Artificial neural network [13] and support vector regression are the two of most widely used prediction models for bearing performance degradation evaluation and residual life prediction [14]. These two models are based on statistical learning theory and data-driven model [15]. Liu et al. used neural network method to predict the performance degradation of rolling bearings [16]. Qian et al. used recurrence quantification analysis and auto-regression model for bearing degradation monitoring and state prediction [17]. Shen et al. used Support Vector Regression (SVR) and statistical parameters of wavelet packet paving to diagnose faults of rotating machinery [18]. Wang et al. used two novel mixed effects models to predict the performance of rolling element bearings [19]. Zhang et al. used SVR to achieve bearing remaining life prediction [20]. Ling et al. used Improved Empirical Wavelet Transform-Least Square Support Vector Machine (IEWT-LSSVM) and bird swarm algorithm to predict wind speed [21]. However, predicting bearing degradation is difficult owing to the non-linearity of bearing data. Due to the nonlinear mapping capability, the kernel methods have attracted the attention of many researchers in recent years. However, different kernel functions have different characteristics [22]. Choosing different kernel functions is crucial for dealing with different problems [23]. To deal with this method, in recent years, different forms of hybrid kernel functions have been studied. Zhou et al. used LSSVM with mixed kernel function build predictive model [24]; Cheng et al. used mixed kernel function support vector regression for global sensitivity analysis [23]. Wu et al. used mixed-kernel based weighted extreme learning machine for the influence of the imbalance datasets problem [25]. Although the hybrid kernel function is applied to many fields but the parameters of the kernel function have a great influence on the prediction results and these parameters are difficult to decide. In this study, Hybrid Kernel Function-Support Vector Regression (HKF–SVR) is proposed to predict bearing performance degradation. Taking into account the uncertainty of the model parameters, the krill herd (KH) algorithm is then used to optimize the parameters of the model.

The rest of this paper is arranged as follows: The second part provides a brief introduction of the KH algorithm and the HKF–SVR. The third part introduces HKF–SVR for the prediction of bearing performance degradation. The fourth part presents two case studies using the proposed method and other methods. The final part presents the conclusions and acknowledgments.

## 2. Theoretical Background

### 2.1. KH Algorithm

The KH algorithm is a bionic intelligent optimization algorithm for the simulation of krill foraging behavior [26]. The local optimal solution is found by attracting or repelling each adjacent krill, the model is simple and fast. At the same time, the krill herd algorithm has good robustness and faster convergence by using group search, compared with other algorithms. Due to using the Lagrange model, the performance of the algorithm is better than other bionic optimization algorithms [27]. Similar to most intelligent optimization algorithms, the KH algorithm generally uses real-coded methods to generate initial populations randomly. The evolution of particles is influenced by three motion components (neighbor induction, foraging movement and random diffusion). The population is increasingly diversified by crossing or mutating individuals until the set termination conditions are met. The KH algorithm is as follows:

Assume that the position of each krill at time *t* is *x*(*t*) and the position after ∆*t* time is *x*(*t* + ∆*t*). On the basis of the basic theory of the KH algorithm [28], the position update of each krill is affected by three speeds, namely, the speed of movement induced by the surrounding krill $N_{i}$, the foraging speed of the krill individual $F_{i}$ and the random diffusion movement $D_{i}$. Here, $N_{i}$ is expressed as follows:

(1) $$N_{i}=N^{\max}\alpha_{i}+w_{n}N_{i}^{old},$$

where $N^{max}$ is the maximum induction velocity, $a_{i}$ is the induction direction, $\omega_{n}$ ∈ (0,1) is the inertial weight and $N_{i}^{old}$ is the velocity vector of the last induced motion. $a_{i}$ is affected by the surrounding krill and the current optimal particles, as shown in the following formula:

(2) $$\left\{ \begin{array}{l} \alpha_{i}=\alpha_{i}^{local}+\alpha_{i}^{target}\\ \alpha_{i}^{local}=\sum_{j=1}^{NP}{\hat{K}}_{i,j}{\hat{x}}_{i,j}\\ {\hat{K}}_{i,j}=\frac{K_{i}-K_{j}}{K^{worst}-K^{best}}\\ {\hat{x}}_{i,j}=\frac{x_{j-}x_{i}}{\left\|x_{j}-x_{i}\right\|+\varepsilon } \end{array}\right.,$$

where $\alpha_{i}^{\mathrm{local}}$ is the direction of induction by the surrounding krill, $\alpha_{i}^{\mathrm{target}}$ is the direction of induction by the current globally optimal individual, ${\hat{K}}_{i,j}$ is the force of the surrounding krill, ${\hat{x}}_{i,j}$ is the current particle’s orientation to the neighbor and *NP* is the population. $K_{i}$ and $K_{j}$ are the fitness values of the current particle and the neighboring particle, respectively. $K^{\mathrm{worst}}$ and $K^{\mathrm{best}}$ are the fitness values of the worst individual and the optimal individual in the current population, respectively. The krill individual foraging speed $F_{i}$ can be expressed by the following formula:

(3) $$F_{i}=V_{f}\beta_{i}+w_{f}F_{i}{}^{old},$$

where $V_{f}$ is the maximum foraging speed, $\beta_{i}$ is the foraging direction and $\omega_{f}$ ∈ (0,1) is the foraging inertial weight.

(4) $$\left\{ \begin{array}{l} \beta_{i}=\beta_{i}^{food}+\beta_{i}^{ibest}\\ \beta_{i}^{food}=2(1-\frac{I}{I_{\max}}){\hat{K}}_{i,\mathrm{food}}{\hat{X}}_{i,food}\\ X_{food}=\frac{\sum_{i=1}^{N}\frac{X_{i}}{K_{i}}}{\sum_{i=1}^{N}\frac{1}{K_{i}}} \end{array}\right.,$$

where $\beta_{i}^{\mathrm{food}}$ and $\beta_{i}^{\mathrm{ibest}}$ are the directions induced by the best individuals of food and particles themselves; $X_{\mathrm{food}}$ is the position of food in which ${\hat{K}}_{i,food}$ is the influence of food on current particles and ${\hat{X}}_{i,food}$ is the orientation of current food to particles. The random diffusion motion $D_{i}$ is expressed as:

(5) $$D_{i}=D_{\max}(1-\frac{t}{t_{\max}})\delta ,$$

where $D_{\max}$. is the maximum random diffusion velocity and *δ* is the random diffusion direction.

The previous theory indicates that the position update of each krill individual is affected by the above three speeds, namely, the motion of the surrounding krill, the krill’s foraging speed and the random diffusion motion. The speed and position update of the particles are expressed as follows:

(6) $$\frac{dx_{i}}{dt}=N_{i}+F_{i}+D_{i}$$

(7) $$x_{i}\left(t+\Delta t\right)=x_{i}\left(t\right)+\Delta t\frac{dx_{i}}{dt},$$

where Δ*t* represents the time interval.

### 2.2. HKF–SVR

SVR is a well-known method to solve the regression problem [29]. Assume the data set is $\left\{x_{i},y_{i}\right\}$, where *x_i_* is the input sample and *y_i_* is the corresponding output value. Then, SVR can be represented by linear function *f*(*x*) = ω*x* + *b*. The SVR function can be represent by introducing *ε* insensitive loss function:

(8) $$\begin{array}{l} y_{i}-\omega \cdot x_{i}-b\leq \varepsilon \;,\;i=1,2,\cdots ,n\\ -y_{i}+\omega \cdot x_{i}+b\leq \varepsilon \end{array},$$

next we can get the Convex optimization problem by minimizing the $\frac{1}{2}{\|\omega }^{2}\|$.

(9) $$\begin{array}{l} \min\;\frac{1}{2}{\left\|\omega \right\|}^{2}+C\sum_{i=1}^{n}\xi_{i}+\xi_{i}{}^{*}\\ s.t\;\left\{ \begin{array}{l} y_{i}-\omega x_{i}-b\leq \varepsilon +\xi_{i}\;i=1,2,\cdots ,n\\ -y_{i}+\omega x_{i}+b\leq \varepsilon +\xi^{*}{}_{i} \end{array}\right. \end{array},$$

where *C* > 0 is the regularization parameter controlling the punishment degree for the sample beyond the error. the relaxation variables $\xi_{i}\geq 0$ and $\xi_{i}^{*}\geq 0$. According to the optimization conditions, we can obtain the dual problem of the support vector regression machine [30] and satisfy the constraint conditions.

(10) $$\left\{ \begin{array}{l} \max\omega (a,a^{*})=-\varepsilon \sum_{i=1}^{n}y_{i}(a_{i}^{*}-a_{i})-\frac{1}{2}\sum_{i,j=1}^{n}\left(a_{i}^{*}-a_{i})(a_{j}^{*}-a_{j})(x_{i}.x_{j}\right)\\ s.t.\left\{ \begin{array}{l} \sum_{i=1}^{n}(a_{i}-a_{i}^{*})=0\\ 0\leq a_{i},a_{i}^{*}\leq C \end{array}\right. \end{array}\right.$$

where *a_i_* and $a_{i}^{*}$ are the Lagrange multipliers. Finally, the regression function [31] is obtained as follows:

(11) $$f(x)=\sum_{i=1}^{n}\left(\alpha_{i}^{*}-\alpha_{i}\right)\langle x_{i}\cdot x\rangle +b^{*}.$$

Different kernel function have different effects on the predicted results [32]. Before the kernel function is constructed, the mapping of input space to feature space must be known. However, if we want to know the mapping of input space to map space, the distribution of data in the input space should be clarified. In most cases, the specific distribution of acquired data is consistently unknown. Thus, constructing a kernel function that fully conforms to the input space is generally difficult. Well-known kernel functions include linear, polynomial, radial basis and sigmoid kernel functions [33]. The linear kernel function is mainly used for linear problems and it has some merits, such as few parameters, fast calculation speed and improved effect for linear separable data. The polynomial kernel function is a global kernel function with many parameters. The radial basis kernel function is a locally strong kernel function that maps a sample into a high-dimensional space and the most widely used of all kernel functions; it has fewer parameters compared with the polynomial kernel function [34]. Thus, the radial basis kernel function is used in most cases.

In this study, a hybrid kernel function is proposed for support vector regression and the model parameters are optimized by the KH algorithm. For the rolling bearing performance degradation prediction, the polynomial and Gaussian kernel functions are selected to construct the hybrid kernel function for SVR and the parameters and the hybrid coefficient of the hybrid kernel function are optimized by the KH algorithm. The constructed HKF–SVR is shown as follows:

(12) $$K_{h}=\lambda K_{poly}+(1-\lambda )K_{rbf},$$

where $K_{\mathrm{poly}}$ is polynomial kernel function, *λ* ∈ (0, 1) is a hybrid coefficient, $K_{\mathrm{rbf}}$ is the radial basis kernel function and $K_{h}$ is a hybrid kernel function.

**Figure 1:**
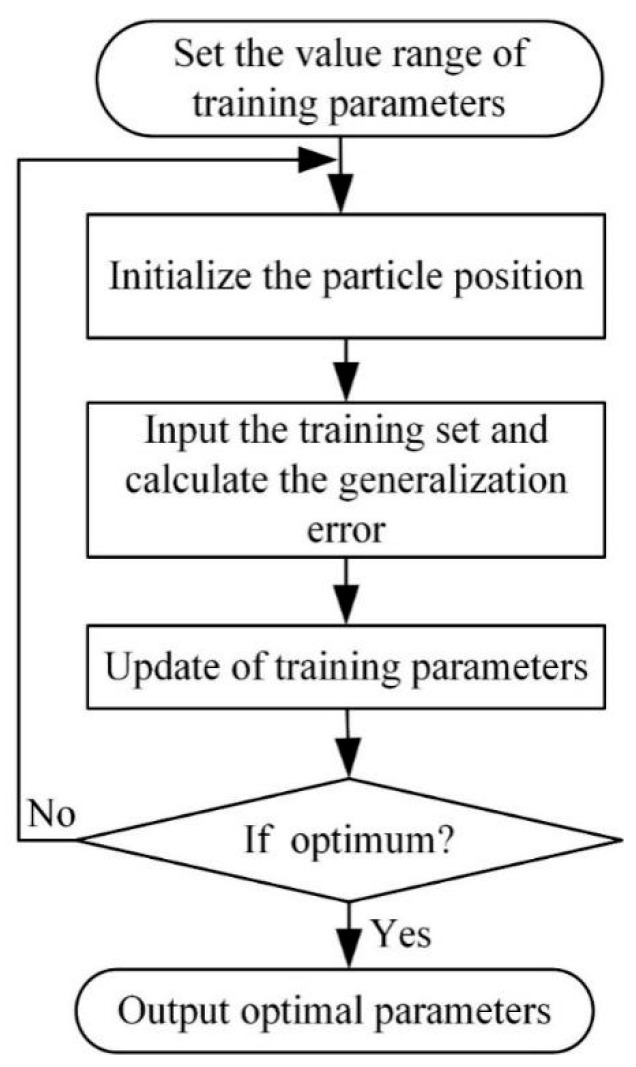
Flow chart of parameters optimization by Krill Herd (KH) algorithm.

The parameters of HKF–SVR include the polynomial kernel function’s highest degree *d*, a gamma1 parameter, the coef0 of the kernel function, the gamma2 parameter of the RBF kernel function, a penalty coefficient *c* and a hybrid coefficient *λ*. The error between the real and predicted values is used as the objective function. The specific parameters and ranges are shown in the Table 1 below. The optimization flow chart of the above parameters by KH algorithm is shown in Figure 1. The specific process is described as follows:

(1) The number of iterations *t*, the number of krill and the maximum number of cycles are initialized.
(2) The value range of parameters is set and the set of parameters is randomly generated as the initial position.
(3) Particle motion and generalization error calculation are conducted.
(4) If the error at a given moment meets the requirements or reaches the number of iterations, Step 7 is performed.
(5) The number of iterations *t* = *t* + 1.
(6) The current particle position and velocity are updated on the basis of Equations (6) and (7), new training parameters are found and then Step 3 is repeated.
(7) The optimal parameters are obtained.

## 3. HKF–SVR Method for Bearing Performance Degradation Prediction

In most real industrial applications, bearings in pairs or even more are mounted on the same shaft. For example, as shown in Case I, there are four bearings on one shaft. So, in this situation, the vibration signal acquired by the sensor mounted on each bearing block will be mixed by the signals propagated through the shaft from the other three bearings. In this study, our recently developed KJADE algorithm is studied on this issue. KJADE is a combination of kernel method and the traditional JADE algorithm. Through the kernel method the latent feature vector can be extracted in the high-dimensional feature space. On the other hand, due to the super blind source separation ability of the JADE algorithm, the correlative feature that could reflect the health status of the monitored bearing can be extracted. The integration evaluation factor of SS is then employed to further evaluate the performance degradation in real time. After that, considering the ability of mixed kernel function to deal with nonlinear problems and the regression prediction ability of SVR, the HKF–SVR is proposed to predict bearing performance degradation. Taking into account the uncertainty of the model parameters, the KH algorithm is then used to optimize the parameters of the model.

**Figure 2:**
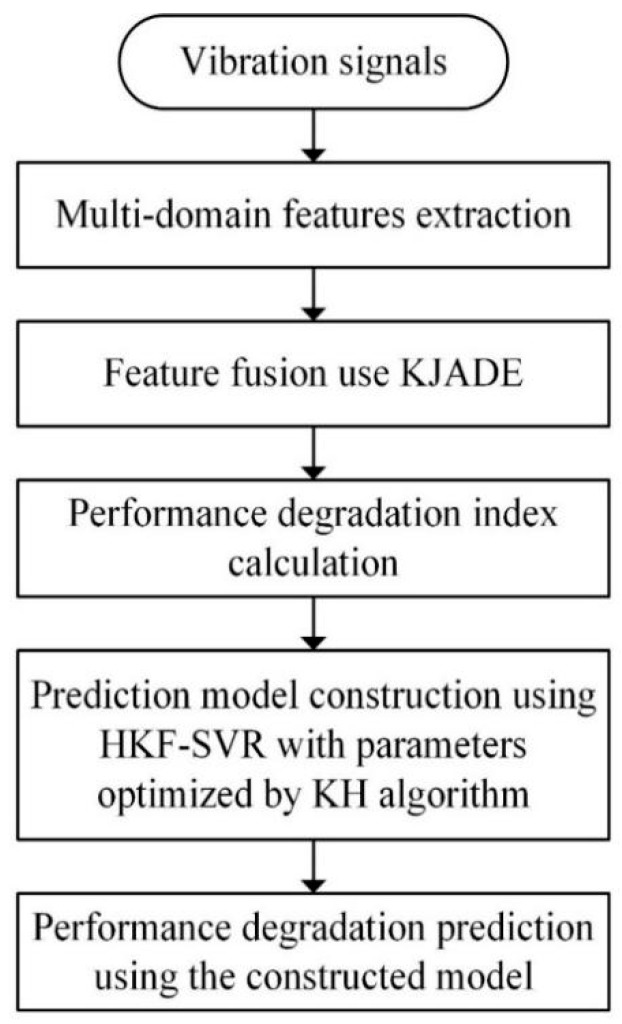
Flow chart of performance degradation prediction by Hybrid Kernel Function-Support Vector Regression (HKF–SVR).

The steps of the whole method are shown in Figure 2 and described as follows:

(1) Multi-domain features extraction. The performance degradation process of bearings has a certain non-linearity. It is difficult for a single feature to accurately reflect the degradation process. In order to comprehensively reflect the bearing state, in this study, eight time-domain features and eight frequency-domain features *F*1-*F*16 shown in Table 2 and Table 3 are extracted from the mixed bearing vibration signal. Where the *F*1-*F*8 stand for mean, root mean square value, square root amplitude, absolute mean, skewness, waveform indicators, pulse indicator and margin index, respectively. *F*9-*F*12 stand for mean frequency, standard deviation of frequency, center frequency, frequency RMS and *F*13-*F*16 stand for the degrees of dispersion or concentration of the spectrum where *s_i_* is a spectrum for *i* = 1, 2, …, *N* (*N* is the number of spectrum lines) and *f_i_* is the frequency value of the *i*-th spectrum line, which indicates the degree of dispersion or concentration of the spectrum and the change of the dominant frequency band. Assume that the sample of the healthy state is *X*, the sample of the current monitoring data sample is *Y*. The two samples are both divided into *n_i_* segments {*X*_1_, *X*_2_, …, *X*_ni_} and {*Y*_1_, *Y*_2_, …, *Y*_ni_} and then the 16 features of each segment are calculated as the original feature vectors {*F^x^*} = {*F^x^*_1,1_, *F^x^*_1,2_, …, *F^x^*_1,ni_; *F^x^*_2,1_, *F^x^*_2,2_, …, *F^x^*_2,ni_; …; *F^x^*_16,1_*, F^x^*_16,2_, …, *F^x^*_16,ni_ }_16 × ni_ *and* {*F^y^*} = {*F^y^*_1,1_*, F^y^*_1,2_, …, *F^y^*_1,ni_; *F^y^*_2,1_, *F^y^*_2,2_, …, *F^y^*_2,ni_; …; *F^y^*_16,1_, *F^y^*_16,2_, …, *F^y^*_16,ni_}_16 × ni_.
(2) Feature fusion using KJADE. In this step, KJADE is used to further extract latent sensitive source features that could accurately reflect the performance degradation of the monitored bearing from the features {*F*^x^} and {*F*^y^} extracted in the previous step. To facilitate visualization of results, the dimension of the latent sensitive source feature vector is set to be 3. So, after this step, the latent sensitive source features are transformed to be {*F*^x^} = {*F*^x^_1,1_, *F*^x^_1,2_, …, *F*^x^_1,ni_; *F*^x^_2,1_, *F*^x^_2,2_, …, *F*^x^_2,ni_; *F*^x^_3,1_, *F*^x^_3,2_, …, *F*^x^_3,ni_ }_3 × ni_ and {*F*^y^} = {*F*^y^_1,1_, *F*^y^_1,2_, …, *F*^y^_1,ni_; *F*^y^_2,1_, *F*^y^_2,2_, …, *F*^y^_2,ni_; *F*^y^_3,1_, *F*^y^_3,2_, …, *F*^y^_3,ni_ }_3 × ni_.
(3) Performance degradation index calculation. The integration evaluation factor of SS between the {F^x^} and {F^y^} obtained in the previous step is calculated as the comprehensive performance degradation index. First, the between-class scatter matrix is calculated as follows:
  (13) $$S_{b}=\sum_{i=1}^{C}p_{i}||m_{i}-m|{|}^{2}.$$
  Then, the inter-class scatter matrix is calculated as follows:
  (14) $$S_{w}=\sum_{i=1}^{C}p_{i}\frac{1}{n_{i}}\sum_{k=1}^{n_{i}}||x_{k}^{i}-m_{i}|{|}^{2},$$
  where *C* is the number of categories, $m_{i}$ is the feature mean in category *i*, *m* is the mean of the entire feature sample. Finally, the *SS* is calculated using the following equation:
  (15) $$SS=trace(S_{b}/S_{w}).$$
  After this step, the *SS* that standing for the performance degradation index of the current monitored data sample can be obtained.
(4) Prediction model constructed through HKF-SVR. In practical engineering application, after continuous monitoring for a period of time, a continuous monitoring vibration data can be obtained. One SS value corresponding to each monitoring moment can be obtained by using steps 1–4 and the performance degradation prediction model can be conducted by using HKF-SVR.
(5) Performance degradation prediction using the constructed model. The performance degradation of the next moment can be predicted using the constructed model obtained in the previous step. Meanwhile, the model is updated in real time with the current and historical data.

## 4. Case Studies

### 4.1. CASE I

**Figure 3:**
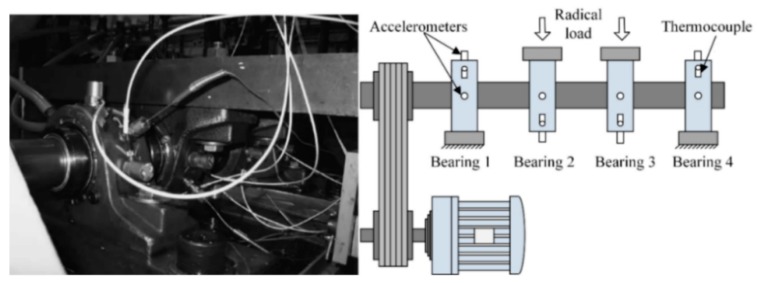
Experimental setup.

In this case, the full-life-cycle bearing vibration signals provided by the Intelligent Maintenance System (IMS) Center of the University of Cincinnati are analyzed using the proposed method. The experimental platform is shown in Figure 3. The four Rexnord ZA-2115 bearings are mounted on the same shaft. The rotational speed of the experimental shaft was maintained at 2000 rpm, the radial load was 6000 lbs., the sampling frequency was 20 kHz and the data length was 20,480 points. The PCB 353B33 quartz sensor was mounted in the horizontal and vertical directions of each bearing and data were collected by the NI data acquisition card DAQ6062E. The acquisition interval between each signal was 10 min.

**Figure 4:**
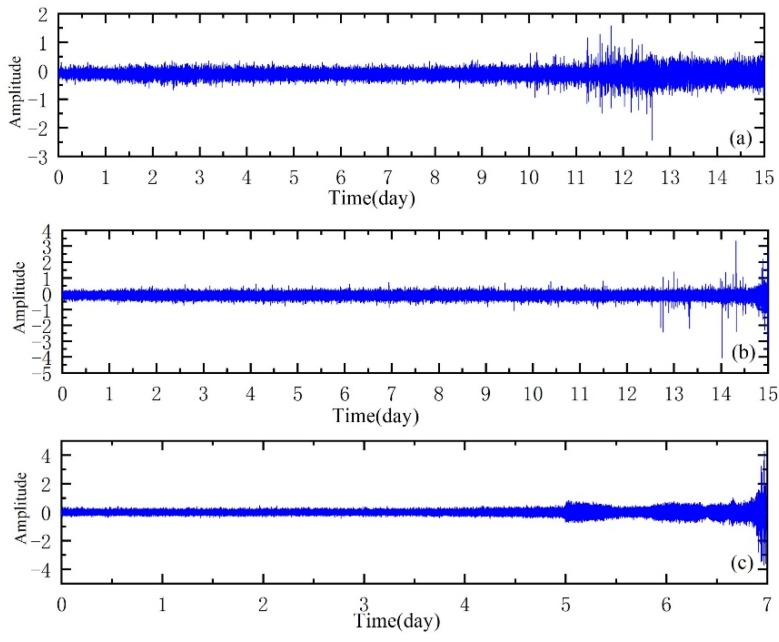
Whole-life vibration signals of bearings.

The entire experiment was completed in three groups. In the first group of experiments, 2156 documents were obtained intermittently. The inner ring of bearing 3 was damaged and the rolling elements of bearing 4 were damaged due to bearing disassembly. In the second set of tests, a total of 984 documents were collected and the outer part of bearing 1 was faulty. In the third set of tests, 4448 documents were obtained and the outer ring fault occurred in the third bearing. Bearings 3 and 4 in the first group of experiments and bearing 1 in the second group of experiments were analyzed. As shown in Figure 4, (**a**) is the full-life-cycle vibration signal of bearing 3, (**b**) is the full-life-cycle vibration signal of bearing 4 and (**c**) is the full-life-cycle vibration signal of bearing 1.

The lifetime data with inner-ring fault, roller fault and outer-ring fault are shown in Figure 4a–c, respectively. The first sample is taken as a healthy sample and the subsequent samples are analyzed as the current monitoring samples. During the analysis, 16,800 points are taken for each analysis sample and divided into 30 segments, so n_i_ = 30 in the step. The method in step one of the third part of this paper is used to extract the 16 dimensions features of the original signal, as shown in Table 2 and Table 3 and then KJADE and *SS* are used to calculate performance degradation indicators. Then HKF-SVR was used to construct a prediction model to predict performance degradation at the next moment.

On the basis of the method proposed in Section 3, the hybrid kernel function of the support vector regression machine was constructed using polynomial and radial basis kernel functions. After the model was established, the parameters of the model were optimized by the KH algorithm. The initial parameters are shown as follows: an initial population of 20, five iterations, a maximum cycle number of 20, a maximum induction velocity *N*^max^ = 0.01, a maximum random diffusion velocity *D*^max^ = 0.005 and a maximum foraging speed *V_f_* = 0.02. These parameters are the optimal parameters obtained through comparative testing. Finally, the HKF–SVR model was obtained to predict the degradation trend of bearing performance.

In this study, the root mean square error (RMSE) was used to evaluate the pros and cons of the method and the results were compared with those of the traditional support vector regression method. The calculation formula of the RMSE is as follows:

(16) $$RMSE=\sqrt{\frac{1}{n}\sum_{i=1}^{n}{\left(y_{i}-{\hat{y}}_{i}\right)}^{2}}.$$

**Figure 5:**
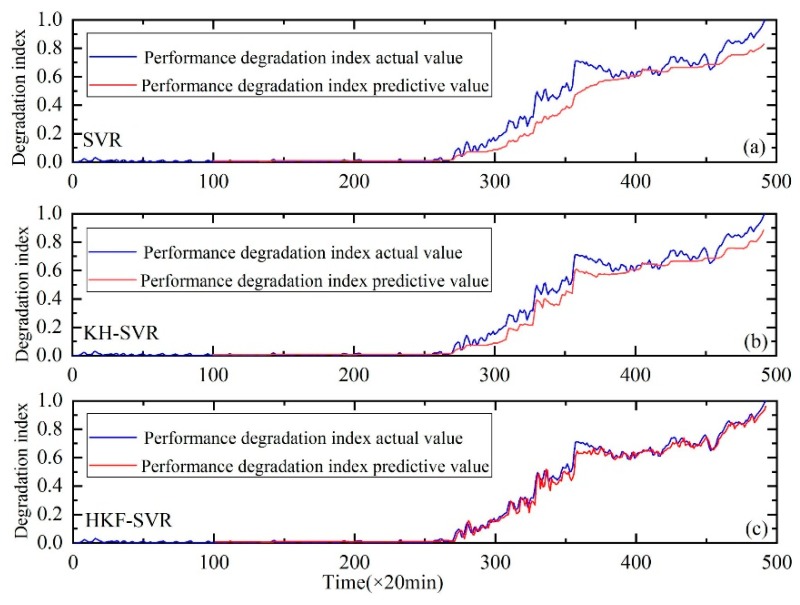
Performance degradation prediction of bearing 1. (**a**) is the result of SVR, (**b**) is the result of SVR with parameters optimized by the KH algorithm and (**c**) is the result of HKF–SVR with parameters optimized by the KH algorithm.

**Figure 6:**
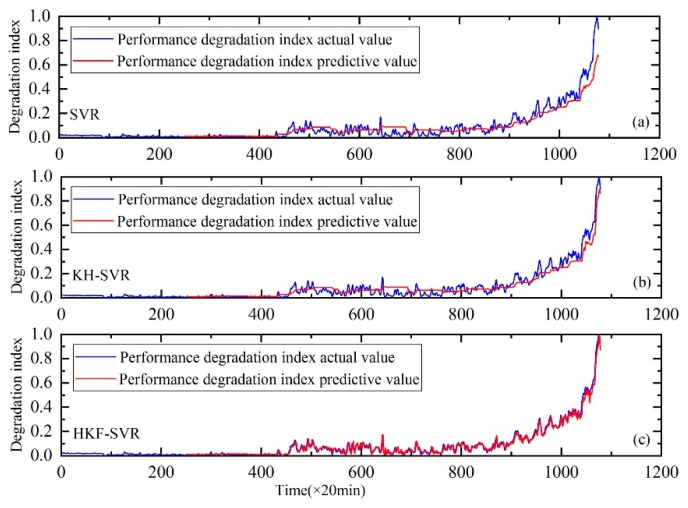
Performance degradation prediction of bearing 3. (**a**) is the result of SVR, (**b**) is the result of SVR with parameters optimized by the KH algorithm and (**c**) is the result of HKF–SVR with parameters optimized by the KH algorithm.

**Figure 7:**
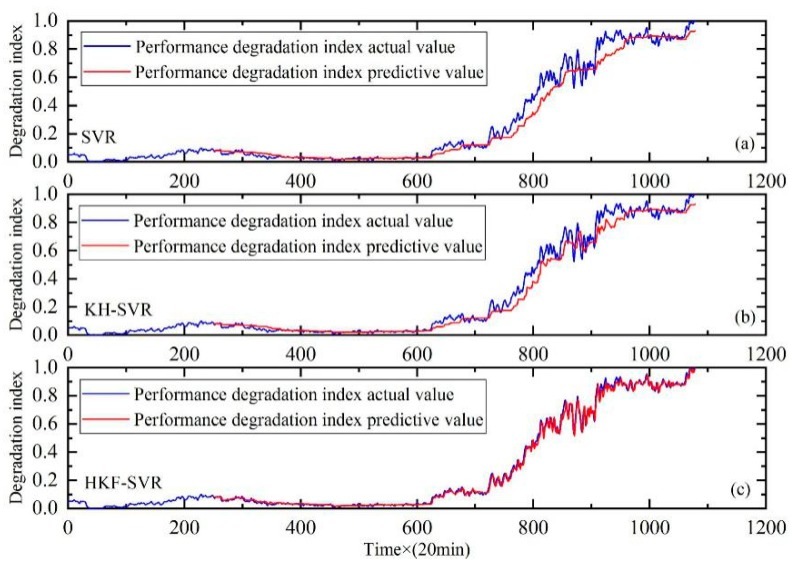
Performance degradation prediction of bearing 4. (**a**) is the result of SVR, (**b**) is the result of SVR with parameters optimized by the KH algorithm and (**c**) is the result of HKF–SVR with parameters optimized by the KH algorithm.

The proposed method was compared with the SVR and support vector regression optimized by the KH algorithm. The results showed that the method could track the performance degradation trend of a bearing; a good prediction result was also obtained. Figure 5, Figure 6 and Figure 7 show the performance degradation prediction graphs of bearing 1 in the second experiment group and bearings 3 and 4 in the first experiment group, respectively. 

The results indicate that the method proposed in this study can effectively predict the performance degradation trend for bearings 1, 3 and 4. Meanwhile, the RMSE values shown in Table 4 indicate that the prediction error of the proposed prediction method is smaller than the other two methods. Thus, this method has advantages over the other two methods.

**Figure 8:**
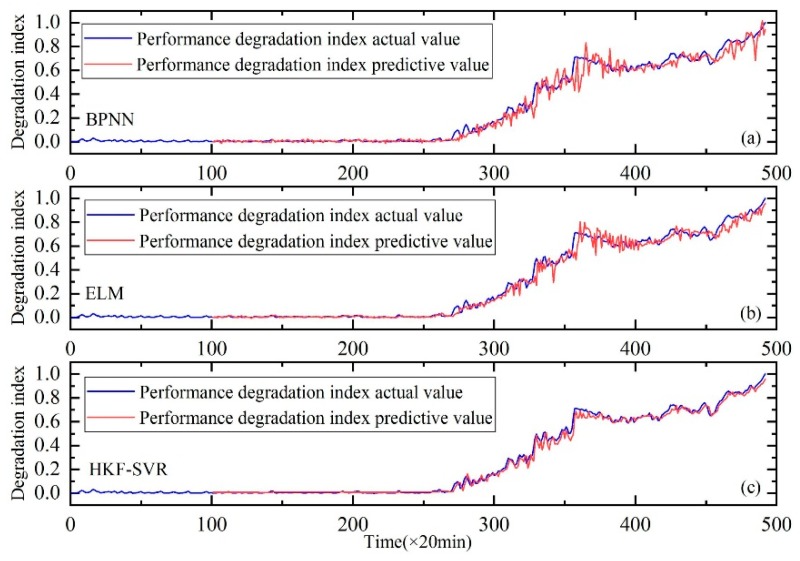
Performance degradation prediction of bearing 1. (**a**) is the result of BPNN, (**b**) is the result of ELM and (**c**) is the result of HKF–SVR with parameters optimized by the KH algorithm.

**Figure 9:**
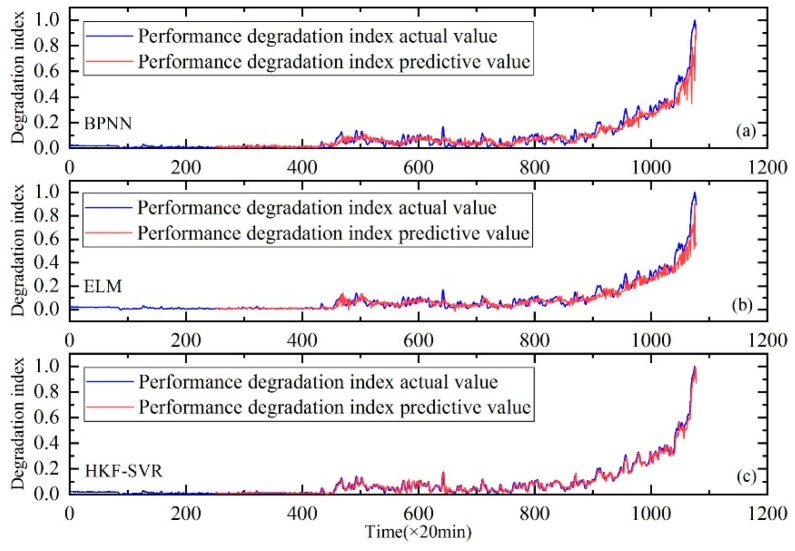
Performance degradation prediction of bearing 3. (**a**) is the result of BPNN, (**b**) is the result of ELM and (**c**) is the result of HKF–SVR with parameters optimized by the KH algorithm.

**Figure 10:**
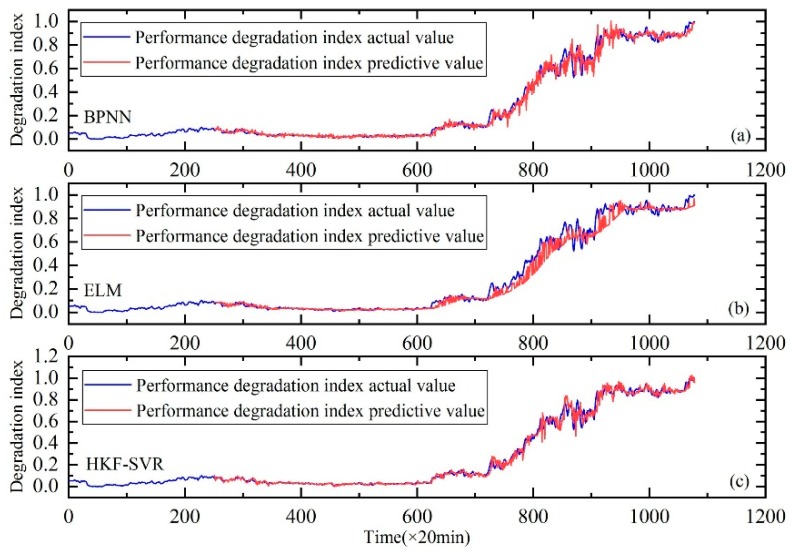
Performance degradation prediction of bearing 4. (**a**) is the result of BPNN, (**b**) is the result of ELM and (**c**) is the result of HKF–SVR with parameters optimized by the KH algorithm.

In order to further prove the validity of the proposed method, the comparison with the Back Propagation Neural Network (BPNN) and Extreme Learning Machine (ELM) are carried out. The results of the comparison are shown in Figure 8, Figure 9 and Figure 10. It can be seen that the prediction results of the HKF-SVR method are more accurate than the others. The RMSE values shown in Table 5 show that the proposed method achieved the minimum prediction error.

**Figure 11:**
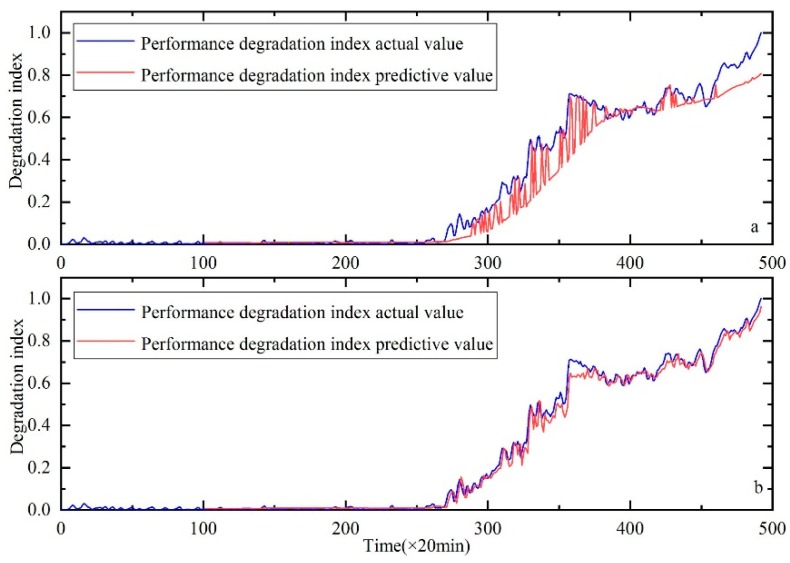
Performance degradation prediction of bearing 1, (**a**) is the result of HKF–SVR with parameters optimized by the GA and (**b**) is the result of HKF–SVR with parameters optimized by KH.

**Figure 12:**
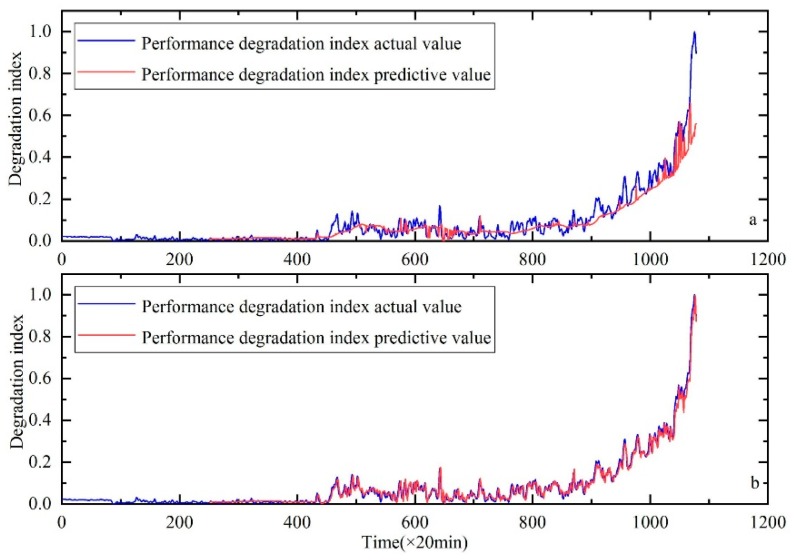
Performance degradation prediction of bearing 3, (**a**) is the result of HKF–SVR with parameters optimized by the GA and (**b**) is the result of HKF–SVR with parameters optimized by KH.

**Figure 13:**
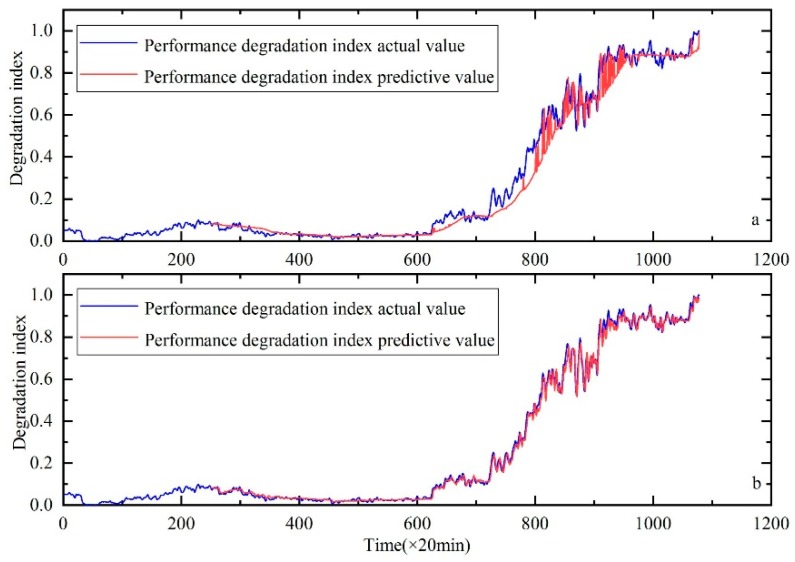
Performance degradation prediction of bearing 4, (**a**) is the result of HKF–SVR with parameters optimized by the GA and (**b**) is the result of HKF–SVR with parameters optimized by KH.

In order to verify the superiority of the KH method, the classic Genetic Algorithm (GA) method is used to optimize the parameters. The comparative results are shown in Figure 11, Figure 12 and Figure 13. RMSE is used to further compare the results of the two methods, as shown in the Table 6. Through the above comparative analysis, it can be seen that the prediction result based on the KH method is more accurate.

### 4.2. CASE II

**Figure 14:**
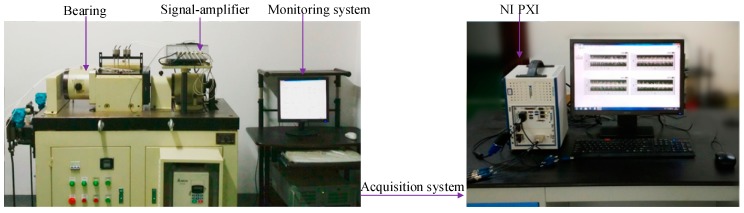
Experimental setup.

The bearing test platform is shown in Figure 14, which includes the ABLT tester and the signal acquisition system based on LabVIEW and NI PXI platforms. The ABLT test machine was produced by the Hangzhou Bearing Test Center, which consists of three systems, namely, control and drive, loading and lubrication systems. The control and drive system enable real-time monitoring of the temperature and vibration signals of the bearings. Four HRB6305 bearing models, which were fixed on the same shaft and driven by an AC motor and connected by a belt, were used in this experiment. The failure of the bearing was accelerated by loading 750 kg in the radial direction of each bearing. After some fatigue tests, three types of faults for the inner ring, outer ring and rolling elements were obtained. Full-life vibration signals were acquired every 5 min by the NI PXI acquisition system. All data were collected at a frequency of 20 kHz and a bearing speed of 3000 rpm.

**Figure 15:**
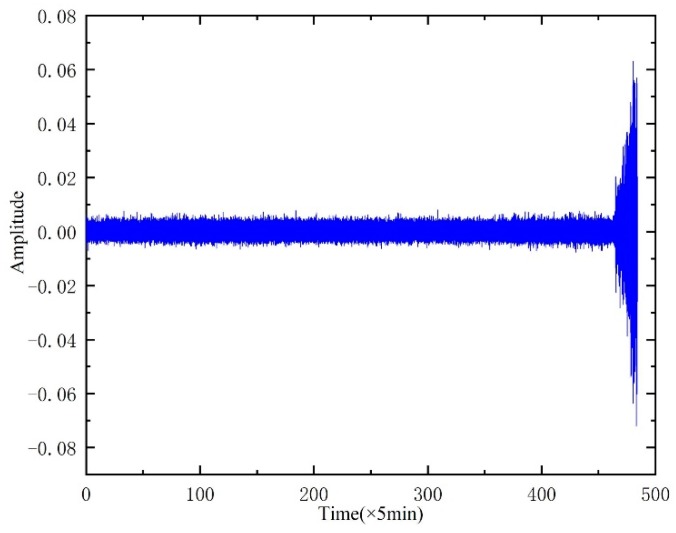
Whole-life vibration signal of bearing.

The full-life original vibration signal of the rolling element is shown in Figure 15.

**Figure 16:**
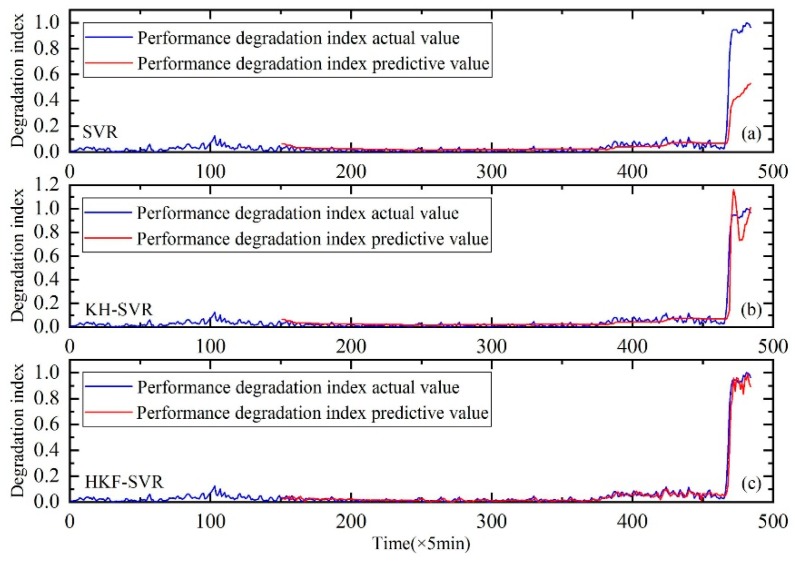
Performance degradation prediction of the rolling bearings, (**a**) is the result of SVR, (**b**) is the result of SVR with parameters optimized by the KH algorithm and (**c**) is the result of HKF–SVR with parameters optimized by the KH algorithm.

Similar to Case I, the features of the time and frequency domains were extracted first. Then, KJADE was used to fuse the original features and the performance degradation index was calculated from inter and between class distances. Finally, the performance degradation of the rolling bearings was predicted using the method proposed in the second part. The results are shown in Figure 16.

The prediction results of SVR, SVR with parameters optimized by KH algorithm and HKF–SVR with parameters optimized by KH algorithm are shown in Table 7, indicating that the method proposed in this study is more effective than the other two methods.

**Figure 17:**
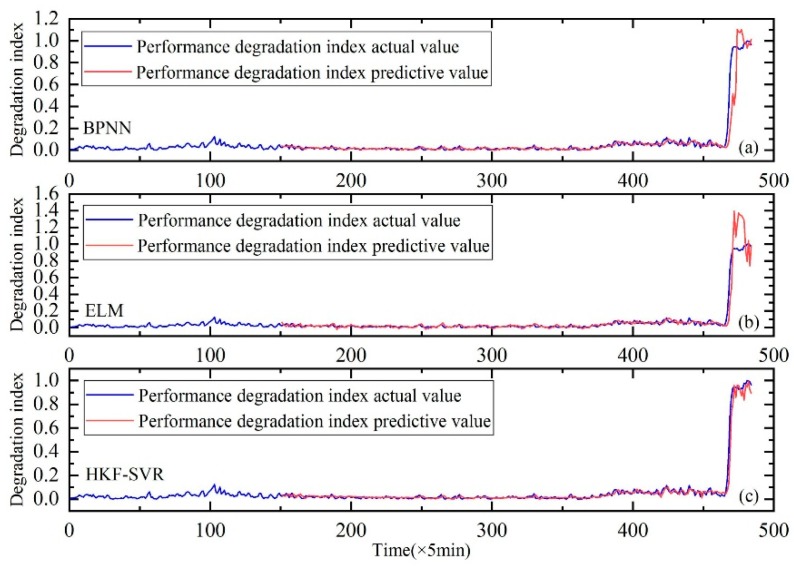
Performance degradation prediction of the rolling bearings. (**a**) is the result of BPNN, (**b**) is the result of ELM and (**c**) is the result of HKF–SVR with parameters optimized by the KH algorithm.

Similar to Case I, the method proposed in this paper is compared with BPNN and ELM. As show in Figure 17, the results also prove the effectiveness of the proposed method.

The RMSE values of the BPNN, ELM and HKF-SVR methods are shown in Table 8.

**Figure 18:**
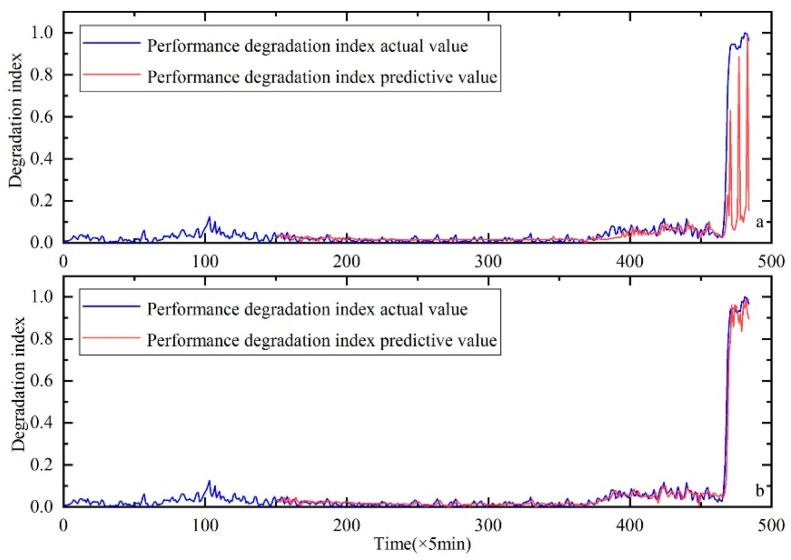
Performance degradation prediction of the rolling bearings, (**a**) is the result of HKF–SVR with parameters optimized by the GA and (**b**) is the result of HKF–SVR with parameters optimized by the KH.

As shown in Figure 18. Meanwhile, RMSE values are shown in Table 9. From the results, we can also see the advantage of the proposed method.

## 5. Conclusions

In this study, the HKF–SVR optimized by the KH algorithm is proposed to predict the degradation of rolling bearing performance for coaxial bearings. It can effectively solve the problem of parameter selection of prediction model. On the other hand, our recently developed KJADE algorithm and SS is studied on performance degradation features extraction for coaxial bearing signals. The proposed method is compared with the SVR, SVR optimized by the KH algorithm, ELM and BPNN. Results have verified the effectiveness and advantage of the proposed method. The proposed method has a good application prospect in life prediction of coaxial bearings.

## Figures and Tables

**Table 1:** Optimized parameters.

Description	Notation
Polynomial kernel function parameter	g1
Polynomial kernel function parameter	coef0
Polynomial kernel function parameter	d
Gaussian kernel function parameters	g2
SVR penalty coefficient	c
Kernel function hybrid coefficient	λ

**Table 2:** Time-domain features.

$F1=\frac{1}{N}\sum_{i=1}^{N}x_{i}$	$F2=\sqrt{\frac{1}{N}\sum_{i=1}^{N}x_{i}^{2}}$
$F3={\left[\frac{1}{N}\sum_{i=1}^{N}\sqrt{\left|x_{i}\right|}\right]}^{2}$	$F4=\frac{1}{N}\sum_{i=1}^{N}\left|x_{i}\right|$
$F5=\frac{1}{N}\sum_{i=1}^{N}x_{i}^{3}$	$F6=\frac{\sqrt{\frac{1}{N}\sum_{i=1}^{N}x_{i}^{2}}}{F4}$
$F7=\frac{max\left(x\right)}{\frac{1}{N}\sum_{i=1}^{N}\left|x_{i}\right|}$	$F8=\frac{\frac{1}{N}\sum_{i=1}^{N}x_{i}^{4}}{{\left(\sqrt{\frac{1}{N}\sum_{j=1}^{N}x_{j}^{2}}\right)}^{4}}$

**Table 3:** Frequency-domain features.

$F9=\frac{1}{N}\sum_{i=1}^{N}s_{i}$	$F10=\frac{1}{N}\sum_{j=1}^{N}(s_{j}-\frac{1}{N}\sum_{i=1}^{N}s_{i}{)}^{2}$
$F11=\frac{\sum_{i=1}^{N}f_{i}s_{i}}{\sum_{j=1}^{N}s_{j}}$	$F12=\sqrt{\frac{\sum_{i=1}^{N}f_{i}^{2}s_{i}}{\sum_{j=1}^{N}s_{j}}}$
$F13=\frac{\frac{1}{N}\sum_{j=1}^{N}(s_{j}-\frac{1}{N}\sum_{i=1}^{N}s_{i}{)}^{3}}{{\left(\sqrt{F10}\right)}^{3}}$	$F14=\sqrt{\frac{1}{N}\sum_{i=1}^{N}s_{i}{\left(f_{i}-F12\right)}^{2}}$
$F15=\sqrt{\frac{\sum_{i=1}^{N}f_{i}^{4}s_{i}}{\sum_{j=1}^{N}f_{j}^{2}s_{j}}}$	$F16=\frac{\sum_{i=1}^{N}f_{i}^{2}s_{i}}{\sqrt{\sum_{j=1}^{N}s_{j}\sum_{k=1}^{N}f_{k}^{4}s_{k}}}$

**Table 4:** Prediction errors comparison.

Method	Bearing 1	Bearing 4	Bearing 3
SVR	0.104	0.082	0.062
KH–SVR	0.079	0.069	0.047
HKF–SVR	0.026	0.027	0.022

**Table 5:** Prediction errors comparison.

Method	Bearing1	Bearing4	Bearing3
BPNN	0.051	0.04	0.047
ELM	0.042	0.055	0.05
HKF-SVR	0.026	0.027	0.022

**Table 6:** Prediction errors comparison.

Method	Bearing1	Bearing4	Bearing3
GA-HKFSVR	0.078	0.052	0.054
KH-HKFSVR	0.026	0.027	0.022

**Table 7:** Prediction errors comparison.

	SVR	KH–SVR	HKF–SVR
RMSE	0.225	0.077	0.035

**Table 8:** Prediction errors comparison.

	BPNN	ELM	HKF-SVR
RMSE	0.067	0.065	0.035

**Table 9:** Prediction errors comparison.

	GA-HKF-SVR	KH-HKF-SVR
RMSE	0.163	0.035
